# Screening for Atrial Fibrillation in Sub-Saharan Africa: A Health Economic Evaluation to Assess the Feasibility in Nigeria

**DOI:** 10.5334/gh.893

**Published:** 2021-12-03

**Authors:** M. S. Jacobs, A. M. Adeoye, M. O. Owolabi, R. G. Tieleman, M. J. Postma, M. Van Hulst

**Affiliations:** 1 Department of Health Sciences, University of Groningen, University Medical Center, Hanzeplein 1, PO box 9700 RB, Groningen, NL; 2 Department of Medicine, College of Medicine, University of Ibadan and University College Hospital, PMB 5116, Ibadan, NG; 3 Center for Genomics and Precision Medicine, College of Medicine, University of Ibadan, PMB 5116, Ibadan, NG; 4 Department of Cardiology, Martini Hospital, Van Swietenplein 1, PO Box 9700 RM, Groningen, The Netherlands.; 5 Department of Cardiology, University Medical Center, University of Groningen, Hanzeplein 1, PO Box 9700 RB, Groningen, NL; 6 Department of Clinical Pharmacy and Toxicology, Martini Hospital, Van Swietenplein 1, PO Box 9700 RM. Groningen, NL

**Keywords:** Atrial Fibrillation, Screening, Health economics, stroke prevention

## Abstract

**Background:**

Cardiovascular disease reflects a major burden of non-communicable disease in Sub-Saharan Africa (SSA). Early detection and treatment of atrial fibrillation (AF), as a preventive measure against stroke, is currently not in the scope of the World Health Organization recommendation to reduce cardiovascular disease.

**Objective:**

We hypothesized that screening for AF would be an important approach to determine the true AF prevalence in the general population in African countries and to identify asymptomatic AF patients at risk for stroke to optimize prevention.

**Methods:**

A decision analytic model was developed to study the health-economic impact of AF screening in Nigeria over a life-time horizon. The patient population explored in the model was a population of newly detected AF cases that would be diagnosed with a one-time systematic screening for AF with a single lead ECG device in community health centres across Nigeria.

**Conclusions:**

The health gain per newly detected AF patient (N = 31,687) was 0.41 QALY at a cost of $5,205 per patient with 100% NOAC use, leading to an ICER of $12,587 per QALY gained. The intervention was cost-effective with a 99.9% warfarin use with an ICER of $1,363 per QALY gained. The total cost of a single screening session was $7.3 million for the total screened population in Nigeria or $1.60 per patient screened. Screening for AF to detect AF patients in need for stroke prevention can be a cost-effective intervention in the Sub-Saharan region, depending on type of anticoagulant used and drug costs.

## Research in Context

### Evidence from before this study

Non-communicable diseases are an increasing health burden in Sub-Saharan Africa (SSA). Cardiovascular disease reflects a major part of this problem, with strokes and heart attacks by far being the most common causes of cardiovascular death. AF prevalence seems to be lower in the African region, most likely due to under-reporting. Under-reporting may be caused by limited access to health care, lack of routine ECG monitoring and limited patient surveillance in Africa.

### Added value of this study

This study showed that screening for AF in SSA can be a cost-effective intervention to detect new AF cases. Cost-effectiveness depends on the type of anticoagulant used and drug costs. The cost-effectiveness is robust in different population setting and within a wide range of AF prevalence assumed.

### Implications of all the available evidence

The World Heart Federation (WHF) has already suggested opportunistic screening of patients 65+ years using pulse palpation. This study showed that population-based screening, based on an age criterion, can be cost-effective when using a simple single-lead ECG device for AF detection. Systematic AF screening should be incorporated in the health care structure to avert future health care costs, especially costs related to stroke.

## Introduction

Non-communicable diseases are an increasing health burden in Sub-Saharan Africa (SSA) [1,2,3,4]. Cardiovascular disease reflects a major part of this problem, with strokes and heart attacks by far being the most common causes of cardiovascular death [1,5,6]. Early detection and treatment of atrial fibrillation (AF), is not part of the total risk approach that the World Health Organization recommends to prevent cardiovascular disease [7]. However, AF increases the risk of stroke about 5-fold and it is associated with a 1.5–2.0-fold increase in all-cause mortality [8]. In a recent review, we concluded that AF prevalence seemed to be lower in the African region, most likely due to under-reporting [9]. Under-reporting may be caused by limited access to health care, lack of routine ECG monitoring and limited patient surveillance in Africa. AF is asymptomatic in a considerable percentage of the AF patients and may remain undetected in daily practice. Stroke has a high impact on health care expenditures and is there with an interesting target for preventive interventions. The European cardiology (ESC) guideline recommends screening for AF in people aged 65 years and older [8]. In African countries the screening approach could be different in terms of target population due to differences in AF characteristics, amongst others the younger age in which AF becomes apparent, and a diverse health care setting. The World Heart Federation (WHF) has developed a roadmap for nonvalvular AF [10]. The WHF suggests two strategies for screening: pulse palpation for patients presenting with AF symptoms or opportunistic screening of patients 65+ years, also with pulse palpation. We hypothesized that screening for AF would be an important approach to determine the true AF prevalence in the general population in African countries and to identify asymptomatic AF patients at risk for stroke to optimize prevention. Early detection of AF can help to identify patients that are eligible for stroke prevention, irrespective of the occurrence of symptoms.

Here, we conducted a health economic evaluation to assess the feasibility of AF screening in SSA with Nigeria as a case study. The evaluation was set up to determine if the costs of screening and the consequent stroke prevention strategy initiated would be a cost-effective intervention or even a cost-saving strategy.

## Methods

### Design and setting

A decision analytic model was developed to study the economic impact of a single session AF screening over a life-time horizon. The patient population explored in the model was a population of newly detected AF cases that would be diagnosed with systematic screening for AF with a single lead ECG device in community health centres across Nigeria. The MyDiagnostick® was the base-case screening method in this evaluation since it is simple to use and not too expensive. Pulse palpation is the recommended AF screening method by the World Heart Federation [10]. We chose this single-lead ECG device for our analysis since it is easy to use and it has a higher sensitivity/specificity (100%/95.9% MyDiagnostick® versus 92%/82% for pulse palpation) [11,12]. Pulse palpation is more time consuming and would require an ECG to confirm the diagnosis and therefore puts a higher burden on health care resource use and costs. Pulse palpation is considered as an alternative scenario (see Method section). The evaluation only focuses on non-valvular AF and not on valvular AF. If AF is mentioned hereafter, non-valvular AF is meant unless explicitly mentioned otherwise.

A decision tree incorporated all relevant variables in the screening procedure and this decision tree served as the input for the Markov model.

**Figure 1:**
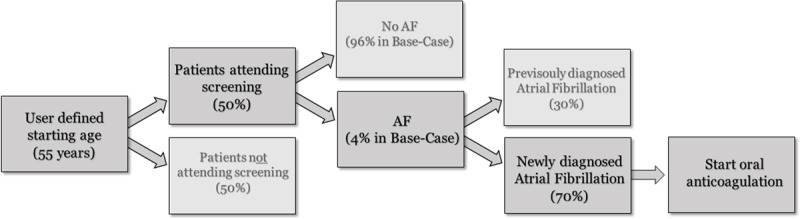
Decision tree for the atrial fibrillation screening procedure.

The decision tree (Figure 1) started with a hypothetical cohort of all people aged ≥ 55 years in Nigeria, a total population of 9,053,294 people. The age of 55 years was chosen as a base-case age, because the 65 years old threshold as described by the ESC was assumed to be too high for a region with a lower life expectancy and appearance of AF at a younger age. Subsequently, a distinction was made between people attending and not attending the screening session. AF prevalence was age-category dependent and the respective prevalence percentages were applied to determine the number of patients with newly diagnosed AF and the number of patients that already had a confirmed AF diagnosis. In the base case analysis, the AF prevalence was set on 1% for the 55+ year old population, 50% of the patients were assumed to attend the screening session and 70% would have unknown AF. The 1% AF prevalence is in line with AF prevalence described in the previously published systematic review [9]. The modelled cohort in the screening group only consisted of newly diagnosed AF patients. The model did not differentiate for type of AF (paroxysmal, persistent or permanent). Although there are studies that show differences in stroke risk for paroxysmal vs persistent or permanent AF, other studies do show an increased or comparable stroke risk for paroxysmal AF comparable with the other AF types [13,14,15,16]. The duration of the episode or burden of the arrhythmia might also determine the stroke risk [17].

Patients were followed in 6-month cycles lifelong until death using a Markov Model approach. In the base-case, anticoagulation therapy with a NOAC (apixaban, dabigatran or rivaroxaban equally distributed) or dose-adjusted warfarin (case) was compared to no treatment (base). All patients with newly detected AF in the case group initiated oral anticoagulation, regardless of the stroke risk. Rate or rhythm control were not incorporated, as these were assessed to be not relevant for stroke risk. Stroke risk was not based on a calculated stroke risk but based on the stroke risk from a real-world study [18]. The yearly stroke risk in this study was 5,4% per year (corrected for death before hospitalization), which corresponded to a CHA_2_DS_2_-VASc >3.

**Figure 2:**
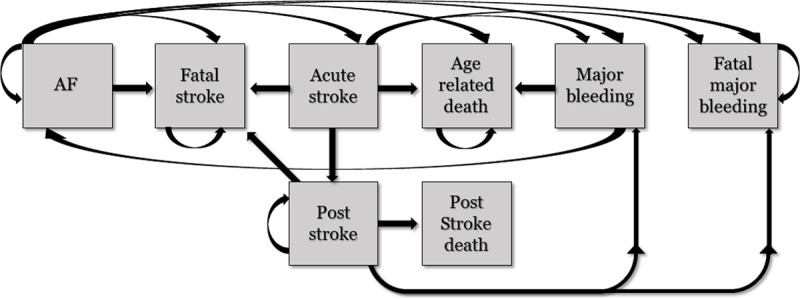
Health states included in the Markov model evaluating life time events in newly diagnosed atrial fibrillation patients. All patients start in the AF state (left).

In the no-screening group, patients were only considered as newly diagnosed AF after having a stroke, therefore AF was assumed not to be detected during daily clinical practice. Assuming the above-mentioned average stroke risk, we assumed that all patients in the base group would initiate oral anticoagulation after a stroke. Data to assess stroke risk scores for the Nigerian population (CHA_2_DS_2_-VASc) was not available and therefore the above-mentioned real-world stroke risk was used. Therapy persistence was assumed to be 70% in the first six months and 60% after one year. Persistence was assumed to be similar for both treatment options. The efficacy of stroke prevention was assumed to remain constant over time. The following health states were included in the base case: (Stable) AF – meaning no event or recent event, Stroke (ischaemic stroke or intracranial bleeding; 67% mild-moderate and 33% moderate-severe to severe), Fatal Stroke, Post Stroke, Post Stroke Death, Major Bleeding (extracranial), Fatal Major Bleeding. Age-related death, Fatal Stroke, Fatal Major Bleeding were incorporated as absorbing states. All major extracranial haemorrhages were assumed to be GI haemorrhages. All patients that experienced a stroke moved to the matching post-event phase after one 6-month cycle if they did not die due to stroke complications or death due to age. The phases ‘Age related death,’ ‘Fatal major bleeding’ and ‘Post stroke death’ were all absorbing end states. The model structure is illustrated in Figure 2.

Costs and effects were reflected from the health care perspective (direct health care costs). The model was developed in Microsoft Excel 2016 software (Microsoft® Inc.). Health gains and costs were discounted by 4%. All unit costs were converted to costs for 2018 in US Dollars. All event probabilities, utilities, costs and other relevant assumptions or input parameters, including their references, are listed in the Supplementary Material.

### Event probabilities

The probability of clinical event outcomes was based on several studies. If country specific information was not available, European data appropriateness was assessed and, if appropriate, this was applied in the model. The 12-month stroke rate was derived from a study in Kenyan AF patients and recalculated into a 6-month probability [19]. Fatal stroke and post stroke death were modelled separately and derived from 30-day case fatality in Nigerian stroke patients [20]. Data on bleeding events was not available for Nigeria. The bleeding rate from a clinical study in AF patients using placebo was used as the base-case bleeding probability and 10% of the major bleedings were assumed to be lethal [21]. Event probabilities are summarized in Table 1.

**Table 1:** Event probabilities per 6 months for major health states included in the Markov model.

Event	6-month probability	Reference

Stroke (Ischemic stroke and intracranial haemorrhage)	0.0108	Temu et al. [19]
Stroke acute death	0.0023	Alkali et al. [20]
Post stroke death	0.1751	Alkali et al. [20]
Major bleeding	0.0013	Hart et al. [21]
Major bleeding acute death	0.0001	Hart et al. [21]
All-cause mortality	Age-dependent	Statistics Nigeria (23)

The clinical event probabilities for patients with AF without stroke prevention were based on pooled trial data from a meta-analysis [21]. Relative risk reduction was assumed to be equal for NOACs and warfarin in the base-case analysis (see Table 2) [22]. The only difference between a NOAC versus warfarin (VKA) treatment was based on the treatment costs and the absence of INR monitoring with NOACs; event probabilities were the same for NOAC vs warfarin in the base-case.

**Table 2:** Relative risk in events probability in patients using oral anticoagulation.

Event	Relative Risk Reduction

Stroke	0.38
Bleeding	0.74
Mortality	2.40

The mortality rate for the simulated population was adjusted for age by increasing the age-specific mortality rate during a patient’s lifetime, starting at 55 years in the base case analysis [23]. Age-specific mortality was not corrected for the event-specific mortality as a specification of mortality causes was not available for the general population. Likely, it can be assumed that stroke and bleeding mortality is most probable underreported, therewith only having limited impact on overall mortality. Event probabilities were modelled with a beta distribution.

### Utilities

Health-related quality of life measures could not all be determined specifically for Nigeria. Therefore, utilities that were not specific for the country were validated for appropriateness by local health care providers. Stroke severity was determined in a Nigerian study that analysed health-related quality of life. Stroke severity was reclassified from five categories into two: mild-moderate and moderate-severe [24]. The quality of life for a major bleeding was calculated using a disutility of –0.029 during six months as determined in ENGAGE AF TIMI study [25]. Utilities were modelled with a beta distribution and a discount rate of 4% was applied. Utilities used in the health economic model are summarized in Table 3.

**Table 3:** Health states and utilities included in the Markov model.

Health state	Utility	Reference

Atrial fibrillation	0.8430	Sullivan et al. [26]
Acute stroke	0.3280	Baeten et al. [27]
Post stroke	0.5490	Baeten et al. [27]
Major bleeding	0.8140	Wang et al. [25]

### Screening costs

The screening session costs were calculated based on the costs of MyDiagnostick, a single lead ECG device. A price of $275 for one year use was taken into account per device, based on €700 per device with a durability of three years. A cohort of 1,000 health care clinics using one ECG device per clinic were included in the analysis. To cover the total 55+ population in Nigeria, per clinic 4,500 people are estimated to be screened. Every positive ECG reading would be checked by a cardiologist to confirm the AF diagnosis. The specificity of the device was not taken into account since it was known that screening costs had only a marginal input on the total screening costs and specificity of the device is high (96%) [28]. The impact of specificity is indirectly incorporated in a sensitivity analysis exploring the effect of screening costs. The costs of the GP were assumed to be one third of the costs for a single visit to a health center (with beds; int$5.20 per visit).

### Costs for events and treatment

Direct health care costs specific for Nigeria were used if available. When there were no Nigeria-specific costs available, data from other African countries was used to estimate the event costs. The World Health Organization CHOICE tool was used to compute several of the cost components by estimating health care utilization and multiplying by standardized costs [29]. All costs for the events are based on a government hospital (stroke costs) or a secondary hospital (bleeding costs) as the default. Post-event costs were based on WHO-CHOICE costs estimates. The WHO-CHOICE estimates represent the basic component of hospital costs, therewith excluding the cost of drugs and diagnostic tests but including costs such as personnel, capital and food costs. The costs for a fatal stroke were 20% higher than a non-fatal stroke. The costs of a fatal major bleeding were 4x the costs of a non-fatal major bleeding. Both assumptions were based on a cost-effectiveness analysis for dabigatran in South-Africa [30]. Costs during the post-stroke phase represent a visit to a secondary hospital once every six months during the remaining lifetime. The costs for a major bleeding are based on a one-week hospitalization in a secondary hospital. The costs for INR monitoring with warfarin are based on a recommended monthly INR check. The costs for anticoagulation were applied during the period in which the patients were alive in the model (life-time costs). All costs for events and the treatment (NOAC, warfarin) are summarized in Table 4.

**Table 4:** Overview of model input cost parameters.

Event costs	Amount (int$) per 6 months	Reference

Acute stroke	$938	Birabi et al [18]
Post stroke	$6	WHO CHOICE, Assumption [29]
Stroke death	$1,126	Birabi et al. [18]
Major bleeding	$195	WHO CHOICE, Assumption [29]
Major bleeding death	$778	WHO CHOICE, Assumption [29]
All-cause mortality	$0	N.A.
**Treatment costs**	**Amount (int$) per 6 months**	

NOAC	$448	Costs for dabigatran (local input)
Warfarin	$17	Based on 5 mg/day (local input)
INR monitoring	$30	Assumption

Costs for health care delivery are listed as international $ units indexed to 2018 using the GDP per capita purchasing power parity (PPP) from the index country compared to 2018. Drug costs and INR monitoring costs are converted from the local currency (NGN) to US dollars using the 2018 exchange rate. All costs were modelled with a beta distribution and a discount rate of 4% was applied.

### Probabilistic sensitivity analysis and scenario analyses

The model was designed to estimate the uncertainty surrounding the cost-effectiveness results by using probabilistic sensitivity analysis (PSA). All model parameters, except for total screening costs, were varied over plausible ranges or, where possible, based on their statistical distribution. If the distribution was unknown, a wide range of –20%/+20% was used. The treatment costs were fixed amounts and therefore not varied in the PSA. The intervention was considered cost-effective at a willingness-to-pay threshold of $2,000/QALY gained which approximates the GDP per capita of Nigeria in 2018 (US$2,028 per capita) [31].

The sensitivity analyses were performed to assess the generalizability of the model results. Four scenario analyses analyzed the effect of population characteristics: the effect of initial age at screening (range 45–65 years), the effect of the prevalence of AF (range 1–7%), the effect of the higher risk reduction of NOACs (19% more reduction of stroke, 10% lower all-cause mortality and 25% more bleeding) [32] and the effect of lower probabilities for developing stroke (50–150% of mean value). All other parameters were kept constant in these scenario analyses and a treatment mix of 100% NOAC use was incorporated. A series of univariate sensitivity analysis were performed to assess the impact of important cost assumptions. The effect of costs was assessed by taking 50% of the mean value as the lower value and 150% of the mean value as the upper value. The costs that were included in these analyses were: stroke costs (acute, post-event and fatal) and single screening session costs. The stroke costs for the three event types were changed simultaneously. Also, the effect of quality of life in the stroke health states was incorporated (50–150% of the mean) by simultaneously changing the utility for acute stroke and the ‘post stroke’ state. A scenario was also calculated for pulse palpation as a screening session. Pulse palpation has an 8% lower specificity than an ECG detection with the MyDiagnostick (92% vs 100% specificity), the screening-detected patients on oral anticoagulation were reduced with 8% and this 8% was added to the first cycle (t = 0) in the screening-detected patients that discontinued treatment. This reflects that pulse palpation would miss 8% of the new diagnosis. For the screening costs, a full health clinic visit was applied per pulse palpation since it’s more time consuming and the higher % false negatives (13.9%; 82% for pulse palpation versus 95.9% for the MyDiagnostick) was added the costs for an ECG for every suspected AF patient and the costs of the cardiologist were raised for the extra ECGs that needed to be analyzed (due to false negatives) [11,12]. All input parameters for the sensitivity analyses are described in Supplementary Material.

### NOAC price threshold analysis

The price of NOACs is fairly high compared to the costs of warfarin therapy, which is the current standard for oral anticoagulation. A price threshold analysis was performed to determine which price would be the turning point from a cost-saving strategy to a cost-effective scenario with higher costs and at which price the treatment would no longer be cost-effective. The costs were varied over a broad range of 1% to 150% of the original country price in the scenario with 100% NOAC use. A broad range for the NOAC price was used because the true cost-effective price was unknown and there was no good estimate for a comparable country. All other parameters were the same as in the base-case scenario.

### Final result outcome measure

Results were calculated as life years and life-time costs for the total patients over the lifetime horizon. Life years corrected for the quality-of-life were reported as ‘quality-adjusted life years’(QALYs) by multiplying time spent in every event state by the quality of life (utility) and summing this over the lifetime horizon. Total costs over lifetime included health care utilization costs, event costs and drug costs. The incremental cost-effectiveness ratio (ICER) is the total costs divided by the total QALYs. Screening would be considered cost-effectiveness at at a willingness-to-pay threshold of $2,000/QALY based on the GDP per capita of Nigeria.

## Results

### Newly detected AF patients and screening costs

The evaluated population consisted of nine million people aged 55 years and older in Nigeria. An AF prevalence of 1% was assumed with 70% having unknown AF. Taking into account the screening attendance rate of 50%, there were 31,687 new AF patients detected with a single screening session. The total cost of a single screening session was $7.3 million for the total screened population or $1.60 per patient screened.

### Base Case

In the base-case, the estimated median survival was 10 years for the no screening scenario versus an estimated median survival of 10.5 years in the screening scenario. The screening scenario (case scenario) was associated with higher costs and higher QALYs, the cost of treatment was 94% of the total costs in the scenario where all patients would be treated with a NOAC. The treatment costs were 62% of the total costs if all patients were treated with warfarin (VKA). The difference in costs for screening vs no screening was $164 million in the 100% NOAC treatment scenario (discounted). Although overall costs were higher in the screening setting due to the treatment costs, $7.8 million was saved on stroke costs compared to the no screening scenario. All results are summarized in Table 5. Per AF patient newly detected, this translates into a health gain of 0.41 QALY at a cost of $5,205 per patient and therefore an ICER of $12,587 per QALY gained.

**Table 5:** Total costs, quality-adjusted life years (QALYs) and incremental cost-effectiveness ratio (ICER) over life-time horizon in 126,746 newly detected AF cases with 100% NOAC use.

Discounted	QALYs	Lys	Costs	ICER

On treatment	232,259	281,456	$192,770,261	
No treatment	219,157	269,191	$27,857,047	
Δ	13,102	12,266	$164,913,214	$12,587/QALY
**Undiscounted**	**QALYs**	**LYs**	**Costs**	**ICER**

On treatment	304,278	372,325	$250,577,298	
No treatment	284,176	350,724	$40,398,697	
Δ	20,102	21,602	$210,178,601	$10,456/QALY

**Figure 3:**
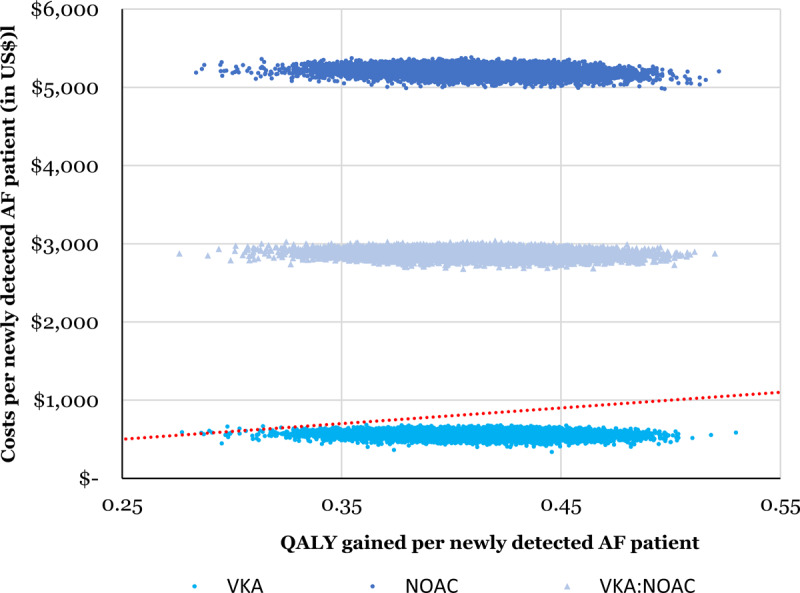
Cost-effectiveness plane showing 10,000 Monte Carlo estimates of incremental costs per patient and benefits per patient of AF screening compared to no screening. Points falling above the linear line have an ICER > $2,000 per QALY gained. Top cloud = 100% NOAC, middle cloud = 50%:50% NOAC:VKA (warfarin), lower cloud = 100% VKA.

Screening with subsequent stroke prevention was cost-effective with a 100% warfarin use. The ICER was $1,363/QALY gained (0.41 QALY gained per newly detected AF patient at a cost of $564 per patient). In the treatment mix scenario with warfarin and NOAC used in a 1:1 ratio, the screening scenario was not cost-effective over the no screening scenario with (ICER $6,975/QALY gained). The results of the probabilistic sensitivity analysis for the three different treatment options are presented in Figure 3. Screening and subsequent stroke prevention was found to be 99.9% cost effective with 100% warfarin use and cost-effective in 0% of the simulations with both 1:1 warfarin (VKA):NOAC use and 100% NOAC at a willingness-to-pay threshold of $2,000 per QALY gained. The results were more sensitive to variation in the parameters influencing quality of life, which is reflected in the flat, long stretched point cloud.

### Sensitvity analyses

Results of the sensitivity analyses in the 100% NOAC treatment regimen are summarized in Table 6. The scenarios related to stroke event probability had the highest impact on the ICER. Adjusting the probability of having a stroke influences both the QALYs as well as the total health care costs related to stroke. The QALYs gained varied over a wide range between 0.18–0.62 QALY gained per newly detected AF patient in the scenario with a lower stroke probability versus the scenario with a higher stroke probability. Varying stroke-related quality of life had an impact on the cost-effectiveness but would not change the cost-effectiveness of screening. AF prevalence had a limited impact on the costs per QALY. Starting age for screening had a high impact on the QALYs gained per newly detected patient, most likely due to the influence on patient survival: 0.71 QALY gained per patient with starting age 45 and 0.20 QALY gained per patient with a starting age of 65 years. The pulse palpation screening scenario was associated with a QALY gain of 0.36 QALY per newly detected patient. Screening versus no screening was not cost-effective in any of the scenario analyses.

**Table 6:** Results of the sensitivity analyses with 100% NOAC treatment.

Scenario description	Lower value ICER (US$/QALY)	Upper value ICER (US$/QALY)

Stroke event probability (50–150%)	$31,063	$7,993
Age (45–65 years)	$10,067	$16,490
Stroke utility (50–150%)	$10,728	$15,226
Pulse palpation for screening (21.0 M€)		$13,280
Relative risk events on NOAC *	–	$14,528
Stroke costs (50–150%)	$12,885	$12,289
AF prevalence (0.5–4%)	$13,087	$12,212
Screening costs (50–150%)	$12,310	$12,864

### NOAC drug cost threshold analysis

A threshold analysis on NOAC drug costs was carried out with screening costs also incorpated in the ICER calculation. Treatment with a NOAC in screening-detected AF patients was not cost-effective with NOAC drug costs of US$448 per six months at a willingness-to-pay threshold of US$2,000 per QALY gained. Screening with subsequent initation of stroke prevention would be cost-effective with NOAC drug costs ≤ US$72 per six months and would be cost-saving with NOAC drug costs ≤US$1 per six months.

## Discussion

Systematic screening for AF is an effective method to identify undiagnosed AF patients. A single screening session could identify over 31,687 new AF patients in Nigeria. The cost-effectiveness of AF screening depended strongly on the type of drug used for subsequent stroke prevention. With warfarin, screening for AF showed a probability of 99.9% to be cost-effective compared to no screening, with a cost of $564 per patient and 0.41 QALY gained. In a 100% NOAC based stroke prevention strategy, screening for AF was not cost-effective at an ICER of $12,587 per QALY gained relative to a GDP per capita based willingness-to-pay threshold of US$ 2,000 per QALY gained. The intervention would be cost-effective if the costs of a NOAC would be $72 per six months or lower, roughly $750 per year cheaper than the current price. The cost-effectiveness was highly influenced by the probability of having a stroke and the starting age for screening. It should be noted that stroke probability was not age-dependent and therefore a lower stroke risk was not linked to a lower starting age for screening. A similar result with regard to stroke probability was found in the evaluation of a single screening session in the Netherlands [28]. The cost-effectiveness was not sensitive to assumptions on AF prevalence, stroke costs and screening costs. This finding with regard to AF prevalence is interesting, because it shows that AF screening can be an effective intervention in LMICs, even if the true AF prevalence of AF would be much lower. The limited sensitivity to AF prevalence is mainly due to the drug treatment costs that are relatively high. A higher AF prevalence also creates higher drug cost expenditures that need to outweigh the healthcare costs due to events. These high drug costs are also the reason why the outcomes were not sensitive to variation in screening costs and stroke costs. A previous review showed a low reported AF prevalence and with this evaluation it is shown that AF screening is then still an interesting strategy. Costs of AF screening are only a very small share (<1%) of the total health care costs over the life-time horizon. The simple single screening session modelled in this paper would only costs $.1.60 per screened patient. The upfront investment of screening is very low, when assuming a 100% use of warfarin the total investment would be around $564 per patient. The total screening session costs of $7.3 million seems to be affordable when comparing this to the GDP of Nigeria which was 448 billion in 2019, screening costs would therefore only be ~0.002% of the GDP. The costs for AF screening with pulse palpation where higher versus the single-lead ECG scenario ($21.0 versus $7.3 million) with a smaller QALY gained (0.36 versus 0.41 QALY per patient). A screening method with a highly sensitive and specific single-lead ECG device therefore seems to be preferred over pulse palpation.

The World Heart Federation (WHF) has suggested opportunistic screening of patients 65+ years using pulse palpation [10]. The WHF acknowledges that early detection and treatment can reduce morbidity and mortality in AF patients. Our results show that screening would be more effective with a lower starting age for screening, both in terms of health gains and costs per patient. These results need to be confirmed by more age-groups specific date on AF and stroke in the African region to confirm that the stroke risk is indeed also increased in younger AF patients. Repetitive screening should also be considered since has been shown that the use of intermittent ECGs can increase the detection rate at least four-fold [33]. Our model was built to analyze a single screening session but repetitive screening seems a cost-effective option to also capture false-negatives, paroxysmal AF patients that were in sinus rhythm during the screening or patient that did not attend the first screening cycle. Opportunistic pulse palpation has a high number of false positives that can results in unnecessary ECGs. An optimal screening method should be cheap to perform with a high specificity and sensitivity. Inexpensive smartphone- or smartwatch-based rhythm monitoring could have a potential application in LMICs to detect AF [34]. The main hurdle for large-scale screening is the low availability of specially trained staff for ECG analysis. Devices with automated ECG detection could overcome this problem and might be a good solution for systematic, or opportunistic, screening in resource low regions. Especially in areas where distances are large and therefore health care is not easily accessible, remote monitoring would be an interesting solution. This not only applies to AF screening, but could also be useful to monitor for example hypertension or heart failure.

In this modelling study for AF screening, stroke prevention with a NOAC was not cost-effective at a WTP threshold of $2,000/QALY. NOACs are at the moment rarely used in low- and middle-income countries while they would undoubtedly would have added value in countries with a less organized health care system. Anticoagulation with VKAs that is not properly and routinely monitored, reflected in the time in therapeutic range (TTR) can lead to ineffective stroke prevention and also an increased risk for bleeding events. There was no TTR estimate available for Nigeria and this variable could therefore not be incorporated in the model structure. TTRs estimates for other African countries, which included Uganda and South Africa, showed a median TTR of only 41% [35]. The efficacy of warfarin is potentially not comparable to a NOAC in LMICs due to expected differences in the TTR. A sub analysis of the RE-LY study showed that the benefits of 150 mg dabigatran at reducing stroke, 110 mg dabigatran at reducing bleeding, and both doses at reducing an ICH versus warfarin were consistent irrespective of centers’ quality of INR as long as the TTR was above 57.1% [36]. With a TTR<57.1%, the effect of ICH was no longer statistically significant for both doses as was the effect of the 110 mg dose on (non-hemorrhagic) stroke and systemic embolism. The base-case assumption in this analysis of no difference in efficacy of the NOACs versus warfarin therefore seems valid. It should be noted that the TTR doesn’t give information on subtherapeutic or supratherapeutic INRs, which is relevant to know to determine the impact on stroke and bleeding, respectively. In the univariate sensitivity analyses we included a higher efficacy for NOACs, meaning a lower risk for stroke (ischemic stroke + ICH), which would be valid if the INR control was suboptimal. As shown in the sensitivity analyses, the stroke probability (ischemic stroke + ICH) had a high impact on the cost-effectiveness of screening with subsequent stroke prevention therapy. Symptomatic status, related to being in sinus rhythm, and rate/rhythm control were not taken into account in the model structure. Several studies have shown that the symptomatic status doesn’t modify the stroke risk and the absence of symptoms or being is sinus rhythm doesn’t necessarily mean that the elevated stroke risk is diminished [37,38].

The WHO publishes a core list of essential medicines [39,40]. In 2015, the WHO committee decided not to add NOACs to the list. In 2019, dabigatran was added to the essential drug list. Generic NOACs could offer a solution to reduce the price level of the new oral anticoagulants. A generic version of dabigatran is already on the Indian market for $26 per month compared to around $70 per month for the originator. With a household capacity to of $89–$243 per month (low-income households and low middle-income countries, respectively), a generic NOAC could be an affordable and cost-effective option for stroke prevention [41]. This health economic evaluation showed that a NOAC can be cost effective in newly detected AF patients at $6/month ($72 per six months of treatment) at a WTP threshold of $2,000/QALY gained, which is considerably lower than the known generic NOAC price. This analysis did not take into account the additional efficacy of NOACs over warfarin in the base-case analysis, wherefore the NOAC price threshold reflects a conservative price.

### Limitations of study

Country and/or region-specific data was limited and could not always be incorporated in the model. Data on true AF prevalence in Sub-Saharan Africa is scarce and therefore the age–related AF prevalence was estimated, also based on data presented in a systematic review [9]. The impact of AF prevalence on the cost-effectiveness was limited and therefore any uncertainty in the true prevalence is not limiting the validity of the results. The efficacy of VKA treatment was based on studies in controlled settings with a good TTR, this might not be fully applicable to resource low regions. Data on quality of life, especially after stroke, was difficult to find. Quality-of-life data was used from high-income countries, which is likely to present an overestimate. Stroke care is less well-arranged in lower income countries and this makes it more likely that quality-of-life of stroke survivors in Sub-Saharan Africa is much lower.

Stroke risk scores were not applied to determine the eligibility for stroke prevention therapy. Reliable data on stroke risk scores in Nigerian AF patients was lacking. With the younger presentation of AF we assumed a shift of the stroke risk, meaning a 55-year old was considered a 65-year old in terms of stroke risk. Stroke risk was not based on a calculated stroke risk but based on the stroke risk from a real-world study [18]. The yearly stroke risk in this study was 5.4% per year (corrected for death before hospitalization), which corresponded to a CHA_2_DS_2_-VASc >3. This implies that on average every patient would be eligible for oral anticoagulation. The model did not include the symptomatic status (paroxysmal versus persistent or permanent), because some studies suggest that the stroke risk is different for paroxysmal AF patients the model might overestimate stroke risk in these patients. A systematic review showed 11.8%–32.1% paroxysmal AF in diagnosed AF patients [9]. Because of the single timepoint screening in this evaluation, it’s likely that the percentage of paroxysmal AF patients would be smaller.

Overall, model input was incorporated in a conservative manner so that the cost-effective would not be overestimated but would rather be underestimated. All model input was validated by local health care providers and researchers. The systematic screening session took into account that 1,000 health care clinics would participate and that they would be able to handle at least 4,500 patients per clinic. We could not fully validate the practical feasibility this assumption, also considering if patients would have easy access to a health care clinic. There was no reliable source to estimate screening attendance, since there is no comparable disease where systematic screening is currently being applied. Although we had to use assumptions for a few parameters, which were validated by health care professionals in Nigeria, the outcome seems robust and representative for the Sub-Saharan region.

## Conclusion

Screening for AF to detect new AF patient in need for stroke prevention can be a cost-effective intervention in the Sub-Saharan region, depending on type of anticoagulant used and drug costs. The cost-effectiveness is robust in different population setting and within a wide range of AF prevalence assumed. Systematic AF screening should be incorporated in the health care structure to avert future health care costs, especially costs related to stroke.

## Additional File

The additional file for this article can be found as follows:

10.5334/gh.893.s1 Supplementary Material. Model input parameters.
