# Supplementary material for: Screening for Atrial Fibrillation in Sub-Saharan Africa: A Health Economic Evaluation to Assess the Feasibility in Nigeria
Source: Glob Heart. 2021 Dec 3;16(1):80. doi: 10.5334/gh.893 (PMC8641527; doi:10.5334/gh.893)
Supplement: Supplementary Material. — Model input parameters. [file gh-16-1-893-s1.pdf]

Supplementary Material

Model input parameters

Variable	Value	Reference
AF prevalence base-case*	1%	1
Starting age for screening*	55 years	1
Population size, according to age threshold	9,053,294 at 55 years	2
Patients attending screening^	50%	3,4 as a basis for the assumption
Patients with unknown AF#	70%	5
Persistence for OAC	70% month 1-6<br>60% onwards	6

** There was no reliable data to assess AF prevalence in Nigeria. There was some data for Sub-Saharan Africa but most studies had a poor study quality due to methods used. We used to average prevalence described in the reference, a systematic review, as the base-case estimate.*

** The 55 years old starting age was used because literature shows that AF appears at an earlier age in Sub-Saharan Africa wherefore numbers for screening from 65+ years old could be a reliable source for the analysis in Nigeria.*

^ The attendance rate was chosen a bit arbitrary since there was no source to use for this, without no data being published on screening attendance for a similar disease area in Sub-Saharan Africa. Only example is the attendance to a colposcopy clinic for women that a positive Pap smear during cervical cancer screening (attendance rate 54%) a self-reported rate of HIV testing within the last 12 months in Sub-Saharan Africa of 46% (mean)

# For Nigeria the % known and unknown AF was turned around (in the reference article it is 30% unknown). This 30% unknown AF reflects a health care system with easy access to health care/a GP or cardiologist and more attention towards AF in general, which is not a appropriate comparison for Nigeria.

References

1 Jacobs et al. Atrial Fibrillation in Africa - An Underreported and Unrecognized Risk Factor for Stroke: A Systematic Review. Global Heart 2019;14:269-279.

2 National Bureau of Statistics. Demographics of Nigeria. 2018; Available at: https://www.nigerianstat.gov.ng. Accessed 11/10, 2019.

3 Knegt et al. Audit of cervical cancer screening and colposcopy attendance in rural South AfricaAfr J Reprod Health 2014;18:70-78

4 Cremin et al. Patterns of uptake of HIV testing in sub-Saharan Africa in the pre-treatment era. Trop Med Int Health 2012;17:e26–e37

5 Kaasenbrood et al. Yield of screening for atrial fibrillation in primary care with a hand-held, single-lead electrocardiogram device during influenza vaccination. Europace 2016:18;1514-152

6 Jacobs MS et al Current Medical Research and Opinion. 2018;34(10):1839–1847

Utilities

Variable	Deterministic value	Lower	Upper	Reference
QALY AF	0,8430	0,7587	0,9273	1
QALY stroke acute	0,3280	0,2952	0,3608	2
QALY post stroke	0,5490	0,4941	0,6039	2
QALY major bleeding*	0,8140	0,7326	0,8954	3

** The quality of life for a major bleeding was calculated using a disutility of -0·029 during 6 months as determined in ENGAGE AF TIMI study*

References Utilities

(1) Sullivan PW et al Preference-Based EQ-5D index scores for chronic conditions in the United States. Med Decis Making 2006;26:410-20.

(2) Baeten S et al. Lifetime health effects and medical costs of integrated stroke services - a non-randomized controlled cluster-trial based life table approach. Cost effectiveness and resource allocation 2010 Jan;8:21

(3) Wang, Ket al. Impact of Spontaneous Extracranial Bleeding Events on Health State Utility in Patients with Atrial Fibrillation: Results from the ENGAGE AF-TIMI 48 Trial. J Am Heart Assoc 2017;6:e006703.

Costs

	Deterministic Value	Lower	Upper	Reference
**Event costs, per event**				
Acute stroke	$938	$570	$1.306	1
Post stroke* (per 6 months)	$6	$3	$8	2
Fatal stroke[#]	$1.126	$684	$1.567	1
Major bleeding^	$195	$118	$271	2
Fatal major bleeding[@]	$778	$473	$1.083	2,3
Drug costs per 6 months				
NOAC	$448	$273	$624	Local author data
VKA	$17	$10	$23	Local author data
INR monitoring	$30	$18	$42	Local author data
**Screening costs MyDiagnostick (price per unit)**				
MyDiagnostick device costs	$275,089 ($275,089)			Estimate
GP costs[+]	$6,277,442 ($1,39)			2
Cardiologist costs	$706,836 ($15.00)			Estimate
Total screening costs	$7,259,367 ($1.60)			Calculated
**Screening costs pulse palpation (per unit)**				
GP costs[++]	$18,832,327 ($3.98)			2
ECG costs	$1,522,202 ($31.75)			Local author data (NGN 3500 for an ECG coverted to US$ with Purchasing Power Parity)
Cardiologist costs	$719,058 ($15.00)			Estimate
Total screening costs**	$21.073.587 ($4.66)			Calculated

** One outpatient visit at a secondary hospital*
# 20% more expensive than an acute stroke
^Hospitalization for one week at a secondary hospital + 10% costs for treatment (average medication costs ratio in stroke)
@ 4x more expensive than non-fatal bleeding based on South African data
+ GP costs for single-lead screening are considered as one third of the costs for a outpatient visit to a health care center with no beds according WHO Choice
++ GP costs for pulse palpation screening are considered as one outpatient visit to a health care center with no beds according WHO Choice
*** Compared to the MyDiagnostick scenario there are 8% of the patients missed in the newly diagnosed AF (8% lower specificity with pulse palpation= 8% false negatives) and 13,9% more false positives (difference in sensitivity). For this calculation, the 8% undiagnosed AF patients are added to the Markov trace with 'treatment discontinuation' in the case base scenario where screening was. This then reflects no diagnosis and thus no anticoagulation. This is needed to make a fair comparison between pulse palpation versus single lead ECG. For the screening costs with pulse palpation the GP and an ECG are the patients with AF + additional percentage which is the difference between the additional false positives (more ECGs to evaluate) subtracted with the false negatives (fewer ECGs to evaluate versus MyDiagnostick scenario). The net results = 13,9% - 8% = 5,9% extra ECGs to perform + to be analyzed by cardiologist.*

References event costs and screening costs

(1) Birabi et al. Cost Burden of Post Stroke Condition in Nigeria: A Pilot Study Global Journal of Health Science 20124:17-22

(2) World Health Organization. World Health Organization CHOosing Interventions that are Cost-Effective (CHOICE) 2014; Available at: www.who.int/choice/cost-effectiveness/en. Accessed 11/19, 2019.

(3) Bergh M, Marias CA, Miller-Jänson H, Salie F, Stander MP. Economic appraisal of dabigatran as first-line therapy for stroke prevention in atrial fibrillation. S Afr Med J 2013;103:241-245

Sensitivity scenario's input

	Deterministic value	Lower value	Upper value	Note
**Age (45-65 years)**	55	45	65	AF% prevalence will change with age adjustment (45 years = 0,3%, 65 years = 4%)
**AF prevalence (0,5-4%)***	1%	0,5%	4%	Age is kept at 55 years
**Relative risk stroke on NOAC**	0,38		0,31	Relative risk reduction is higher for NOACs compared to VKA
**Relative risk mortality NOAC**	0,74		0,67	
**Relative risk bleeding NOAC**	2,40		3,00	
**Stroke costs: Acute stroke**	$938	$469	$1.407	Changed simultaneously, all other values on deterministic
**Stroke cost: Post stroke**	$6	$3	$9	
**Stroke costs: Stroke death**	$1.126	$563	$1.689	Changed simultaneously, all other values on deterministic
**Bleeding costs: major bleeding**	$195	$97	$292	Changed simultaneously, all other values on deterministic
**Bleeding costs: major bleeding death**	$778	$389	$1.167	
**Screening costs**	$7.259.367	$3.629.683	$10.889.050	Only adjust screening costs
**Utility: acute stroke**	0,328	0,164	0,492	Changed simultaneously, all other values on deterministic
**Utility: post stroke**	0,549	0,274	0,823	
**Stroke probability: acute stroke**	0,011	0,005	0,016	Changed simultaneously, all other values on deterministic
**Stroke probability: post stroke death**	0,175	0,088	0,263	
**Stroke probability: acute stroke death**	0,0023	0,001	0,003	
**Pulse palpation for screening**			€ 21.073.587	Decrease treated in "Markov Treated with 8% (difference in sensitivity ECG vs pulse pap) and add this 8% to treatment discontinued in the 'screened' arm. Cost estimate based on additional ECG needed for diagnosis confirmation + extra patient due to positive balance in sum "false positives - false negatives"

* 4% as an upper AF prevalence based on Kaasenbrood et al screening paper.
